# Supplementary material for: Differential reinforcement encoding along the hippocampal long axis helps resolve the explore–exploit dilemma
Source: Nat Commun. 2020 Oct 26;11:5407. doi: 10.1038/s41467-020-18864-0 (PMC7589536; doi:10.1038/s41467-020-18864-0)
Supplement: Supplementary file 1 — Supplementary Information [file 41467_2020_18864_MOESM1_ESM.pdf]

Supplementary material for:

Differential reinforcement encoding along the hippocampal long axis helps resolve the explore-exploit dilemma

Alexandre Y. Dombrovski, Beatriz Luna, Michael N. Hallquist

Contents:

Supplementary Note 1.

Supplementary Table 1. Clusters of voxels significantly modulated by reward prediction errors (RPEs) in whole-brain analyses.
Supplementary Table 2. Clusters of voxels significantly modulated by model-estimated entropy in whole-brain analyses.
Supplementary Table 3. Factor structure of regions responsive to reward prediction error.
Supplementary Table 4. Factor structure of regions responsive to entropy.
Supplementary Table 5. Behavioral effects of hippocampal signals: main and sensitivity analyses, fMRI session.
Supplementary Table 6. Behavioral effects of hippocampal signals: main and sensitivity analyses, replication session.
Supplementary Table 7. Response hazard as a function of time-varying value and uncertainty and their interactions with hippocampal signals, mixed-effects Cox model

Supplementary Figure 1. Posterior hippocampal responses to reward prediction error in the current study but not in prior meta-analyses.
Supplementary Figure 2. Encoding of reinforcement along the A-P axis when separate regressors are used to model the first half versus second half of each run of the clock task.
Supplementary Figure 3. Hippocampal encoding of immediately preceding response time: sensitivity test for multi-level analysis of deconvolved responses.
Supplementary Figure 4. Encoding of reinforcement along the A-P axis when modeled by simultaneous versus separate model-based decision signals in voxelwise analyses.
Supplementary Figure 5. Hippocampal online responses to the global value maximum, sensitivity analysis using a restrictive mask.
Supplementary Figure 6. Hippocampal encoding of reinforcement, sensitivity analysis using a restrictive mask.

Supplementary Note 1

In sensitivity analyses, we ascertained that the doubly dissociable effects of PH vs. AH responses on exploration vs. exploitation remained unchanged after controlling for behavioral variables (trial, contingency, maximum available value, uncertainty, and their interactions), subject-level performance (mean entropy and value) and for interactions between these potential confounds and hippocampal responses (Supplementary Table 5). The addition of each block of variables improved the fit of the model predicting RT but did not change the substantive effects of AH and PH on choice. Sensitivity analyses of the replication session yielded essentially identical results such that PH predicted exploration (RT swing) and AH predicted exploitation (convergence on global value maximum) even after controlling for other behavioral variables (Supplementary Table 6).

Since we used the SCEPTIC RL model to generate RPE and entropy estimates tested against hippocampal activity, we ascertained that our findings were not tautologically explained by other model-derived covariates (e.g. maximum available value) into statistical models predicting behavior. We thus performed "model-free" (i.e., no variables from the SCEPTIC model) behavioral analyses including only RT_{t-1}, trial, last outcome and condition as covariates. In these reduced models, PH RPE still strongly predicted greater exploration (fMRI session, $RT_{t-1} \times$ PH: $t = -12.46$, $p < 10^{-15}$; replication session, $t = -7.32$, $p < 10^{-12}$), particularly following rewards (fMRI session, $RT_{t-1} \times$ last outcome × PH: $t = 5.77$, $p < 10^{-8}$; replication session, $t = 4.27$, $p < 10^{-4}$). In a model without RT_{Vmax}, AH low entropy responses predicted less exploration (fMRI session, $RT_{t-1} \times$ AH: $t = 2.92$, $p = .004$; replication session, $t = 1.30$, $p = .20$), particularly after reward omissions (fMRI session, $RT_{t-1} \times$ last outcome × AH: $t = 2.18$, $p = .029$; replication session, $t = 5.15$, $p < 10^{-6}$). However, adding RT_{Vmax} back to the model revealed that this effect was partly explained by greater exploitation in participants with strong AH responses (fMRI session, $RT_{t-1} \times$ AH: $t = 0.81$, $p = .41$, $RT_{t-1} \times$ last outcome × AH: $t = 2.19$, $p = .028$, $RT_{Vmax} \times$ AH: $t = 2.74$, $p = .006$, -1/trial × $RT_{Vmax} \times$ AH: $t = 2.44$, $p = 0.015$; replication session, $RT_{t-1} \times$ AH: $t = 0.57$, $p = .566$, $RT_{t-1} \times$ last outcome × AH: $t = 5.01$ $p < 10^{-6}$, $RT_{Vmax} \times$ AH: $t = 1.48$, $p = .14$, -1/trial × $RT_{Vmax} \times$ AH: $t = 3.07$, $p = 0.002$). Finally, since participants' hippocampal activity estimates were based on reinforcement, we removed all reinforcement information from the model predicting choices. In this reduced model, our primary effects of interest were still robust, even though the effect of AH on exploitation during the replication session was only evident later in learning (fMRI session, $RT_{t-1} \times$ PH: $t = -9.53$, $p < 10^{-15}$, $RT_{Vmax} \times$ AH: $t = 2.63$, $p = .009$, -1/trial × $RT_{Vmax} \times$ AH: $t = 2.53$, $p = 0.011$; replication session, $RT_{t-1} \times$ PH: $t = -3.97$, $p < 10^{-4}$, $RT_{Vmax} \times$ AH: $t = 0.80$, $p = 0.42$, -1/trial × $RT_{Vmax} \times$ AH: $t = 3.01$, $p = 0.003$).

Given the established role of cortico-striatal networks in reward learning, it was important to ascertain that hippocampal signals predicted behavior above and beyond cortico-striatal signals identified in the literature and our study in particular (Tables S1-S4). Thus, we tested for region and signal specificity of hippocampal effects on exploration/exploitation after controlling for the strength of neural reward signals in other key regions. These included vmPFC expected value responses, dorsal attention network responses to high entropy and cortico-striatal prediction error responses. All effects of hippocampal signals were robust in both the fMRI and replication sessions (fMRI session: $RT_{t-1} \times$ PH: $t = -7.16$, $p < 10^{-12}$, $RT_{Vmax} \times$ AH: $t = 3.57$, $p < .001$, -1/trial × $RT_{Vmax} \times$ AH: $t = 2.07$, $p = 0.038$; replication session: $RT_{t-1} \times$ PH: $t = -5.45$, $p < 10^{-7}$, $RT_{Vmax} \times$ AH: $t = 3.17$, $p = .0015$, -1/trial × $RT_{Vmax} \times$ AH: $t = 2.52$, $p = 0.012$).

Supplementary Table 1. Clusters of voxels significantly modulated by reward prediction errors (RPEs) in whole-brain analyses.

				**Cluster center-of-mass MNI coordinates**		
	**Regions within cluster**	**Number of Voxels**	z_{max}	**x**	**y**	**z**
1	Dorsal anterior cingulate cortex, midcingulate cortex, bilateral fusiform gyrus, bilateral intraparietal sulcus, bilateral supplementary motor area, bilateral cerebellum (Crus I), bilateral precuneus	13641	8.58	0.7	-56.0	15.2
2	Left ventral striatum, right ventral striatum, left putamen, right putamen, bilateral thalamus, left inferior frontal gyrus pars opercularis, left anterior insula, right anterior insula	3803	8.03	-7.4	0.7	4.9
3	Left inferior frontal gyrus pars triangularis	704	5.15	-42.0	37.0	9.2
4	Right inferior frontal gyrus pars opercularis	494	7.58	47.8	10.2	26.6
5	Right inferior frontal gyrus pars triangularis	431	5.14	43.6	38.4	12.6
6	Left anterior insula/frontal operculum	204	5.40	-33.2	21.6	-1.6
7	Right posterior hippocampus	190	7.21	21.8	-34.2	-1.5
8	Left superior occipital gyrus	185	-5.89	-13.9	-97.4	18.2
9	Right superior occipital gyrus	165	-4.53	18.3	-94.8	20.2
10	Left posterior hippocampus	152	5.24	20.8	-36.1	-0.8
11	Right superior frontal gyrus	146	4.43	28.8	4.9	62.0

Note. Clusters of significant voxels were identified based on a voxelwise threshold of $|z| > 3.09$ ($p < .001$, one-tailed) and a cluster threshold of $p < .05$. The cluster thresholds were identified using by randomizing the sign of the residuals of a one-sample *t*-test of the whole-brain RPE analysis using AFNI *3dttest++ -Clustsim*. This procedure does not assume a Gaussian autocorrelation function and determined that clusters > 107 voxels were significant at the whole-brain level. Cluster numbers are given in the order of decreasing size.

Supplementary Table 2. Clusters of voxels significantly modulated by model-estimated entropy in whole-brain analyses.

	Regions within cluster	Number of Voxels	z_{max}	Cluster center-of-mass MNI coordinates x	y	z
	Regions sensitive to low entropy					
4	Left postcentral gyrus	417	-4.53	-38.9	-24.1	61.6
5	Bilateral ventromedial prefrontal cortex	282	-4.39	-6.2	52.5	-5.9
8	Right Rolandic operculum	193	-4.98	51.2	-20.5	16.7
9	Left anterior hippocampus	159	-5.26	-28.3	-16.7	-16.9
10	Left fusiform gyrus	148	-5.02	-22.8	-44.9	-13.8
11	Left Rolandic operculum	132	-4.88	-52.5	-13.3	16.5
	Regions sensitive to high entropy					
1	Bilateral precuneus, bilateral inferior parietal lobule	2435	6.64	10.1	-60.6	54.7
2	Right superior frontal gyrus/frontal eye field	664	5.70	26.6	1.9	56.1
3	Left cerebellum (Crus 1)	440	5.14	-37.6	-55.6	-34.6
6	Left superior frontal gyrus/frontal eye field	263	5.36	-26.0	-2.2	58.1
7	Right rostrolateral prefrontal cortex	208	4.97	33.3	56.6	6.2

Note. Clusters of significant voxels were identified based on a voxelwise threshold of $|z| > 3.09$ ($p < .001$, one-tailed) and a cluster threshold of $p < .05$. The cluster thresholds were identified using by randomizing the sign of the residuals of a one-sample *t*-test of the whole-brain RPE analysis using AFNI *3dttest++ -Clustsim*. This procedure does not assume a Gaussian autocorrelation function and determined that clusters > 117 voxels were significant at the whole-brain level. Cluster numbers are given in the order of decreasing size.

Supplementary Table 3. Factor structure of regions responsive to reward prediction error

	Region	Factor 1	Factor 2
4	Right inferior frontal gyrus pars opercularis	**0.887**	0.006
5	Right inferior frontal gyrus pars triangularis	**0.878**	-0.151
6	Left anterior insula/frontal operculum	**0.740**	0.052
2	Bilateral striatum	**0.645**	0.294
1	Cingulate cortex and precuneus	**0.622**	0.169
3	Left inferior frontal gyrus pars triangularis	**0.603**	0.115
11	Right superior frontal gyrus	**0.582**	-0.09
10	Left posterior hippocampus	-0.04	**0.973**
7	Right posterior hippocampus	0.196	**0.731**

Note. The highest factor loading is printed in bold face. Cluster numbers match Supplementary Table 1.

Supplementary Table 4. Factor structure of regions responsive to entropy

	Region	Factor 1	Factor 2
10	Left fusiform gyrus	**0.830**	0.213
8	Right Rolandic operculum	**0.796**	0.304
11	Left Rolandic operculum	**0.717**	0.298
9	Left anterior hippocampus	**0.710**	-0.066
4	Left postcentral gyrus	**0.708**	0.241
5	Bilateral ventromedial prefrontal cortex	**0.538**	-0.199
1	Bilateral precuneus	0.29	**0.850**
2	Right superior frontal gyrus	0.142	**0.831**
3	Left cerebellum (Crus 1)	0.057	**0.742**
6	Left superior frontal gyrus	0.349	**0.638**
7	Right rostrolateral prefrontal cortex	-0.133	**0.632**

Note. The highest factor loading is printed in bold face. Cluster numbers match Supplementary Table 2.

Supplementary Table 5. Behavioral effects of hippocampal signals: main and sensitivity analyses, fMRI session.

	Dependent variable:			
	RT			
	Main analysis	+ contingency	+ choice uncertainty	+ subject-level performance
	(1)	(2)	(3)	(4)
-1/trial	0.01 (0.01)	-0.01 (0.03)	0.02 (0.03)	0.02 (0.03)
RT(t-1)	0.5 (0.01)***	0.5 (0.01)***	0.5 (0.01)***	0.5 (0.01)***
RT(Vmax, t-1)	0.1 (0.01)***	0.1 (0.01)***	0.1 (0.01)***	0.1 (0.01)***
last outcome: omission vs. reward	-0.2 (0.01)***	-0.2 (0.01)***	-0.2 (0.01)***	-0.2 (0.01)***
Vmax, within-subject	0.01 (0.01)	0.01 (0.01)	0.01 (0.01)	0.01 (0.01)
entropy, within-subject	0.02 (0.01)***	0.02 (0.01)***	0.02 (0.01)*	0.02 (0.01)*
AH low entropy resp.	-0.01 (0.1)	-0.1 (0.2)	-0.1 (0.2)	1.2 (0.6)
PH RPE resp.	-0.03 (0.04)	-0.1 (0.04)	-0.1 (0.04)	-0.2 (0.1)
contingency: CEVR vs. CEV		0.1 (0.05)	0.05 (0.05)	0.1 (0.05)
contingency: DEV vs. CEV		-0.1 (0.04)***	-0.1 (0.04)***	-0.1 (0.04)**
contingency: IEV vs. CEV		0.1 (0.04)***	0.1 (0.04)***	0.1 (0.04)***
uncertainty of last choice			0.03 (0.01)***	0.03 (0.01)***
mean entropy, between-subjects				0.6 (0.1)***
mean Vmax, between-subjects				-0.001 (0.001)
-1/trial * RT(t-1)	-0.1 (0.01)***	-0.1 (0.01)***	-0.1 (0.01)***	-0.1 (0.01)***
-1/trial * last outcome	0.1 (0.01)***	0.1 (0.01)***	0.1 (0.01)***	0.1 (0.01)***
-1/trial * RT(Vmax)	0.01 (0.02)	0.02 (0.02)	0.02 (0.02)	0.02 (0.02)
-1/trial * Vmax	0.01 (0.01)*	0.02 (0.01)*	0.01 (0.01)*	0.02 (0.01)*
-1/trial * entropy	-0.01 (0.01)	-0.01 (0.01)	-0.01 (0.01)	-0.01 (0.01)
-1/trial * AH	-0.01 (0.04)	0.1 (0.1)	0.03 (0.1)	0.03 (0.1)
-1/trial * PH	0.01 (0.01)	0.02 (0.02)	0.02 (0.02)	0.02 (0.02)
RT(t-1) * RT(Vmax)	-0.003 (0.01)	-0.002 (0.01)	0.003 (0.01)	0.004 (0.01)
RT(t-1) * last outcome	-0.4 (0.01)***	-0.4 (0.01)***	-0.4 (0.01)***	-0.4 (0.01)***
RT(t-1) * Vmax	-0.03 (0.01)***	-0.03 (0.01)***	-0.03 (0.01)***	-0.03 (0.01)***
RT(t-1) * entropy	-0.02 (0.01)**	-0.02 (0.01)**	-0.02 (0.01)**	-0.02 (0.01)**
RT(t-1) * AH	0.04 (0.04)	0.02 (0.04)	0.04 (0.04)	0.04 (0.04)
**RT(t-1) * PH**	**-0.1 (0.01)*****	**-0.1 (0.01)*****	**-0.1 (0.01)*****	**-0.1 (0.01)*****
RT(Vmax) * last outcome	0.1 (0.01)***	0.1 (0.01)***	0.1 (0.01)***	0.1 (0.01)***
RT(Vmax) * Vmax	0.1 (0.01)***	0.1 (0.01)***	0.1 (0.01)***	0.1 (0.01)***
RT(Vmax) * entropy	0.01 (0.01)	0.01 (0.01)	0.01 (0.01)	0.01 (0.01)
**RT(Vmax) * AH**	**0.1 (0.04)*****	**0.1 (0.04)****	**0.1 (0.04)****	**0.1 (0.04)****
RT(Vmax) * PH	0.01 (0.01)	0.01 (0.01)	0.01 (0.01)	0.01 (0.01)
last outcome * Vmax	-0.02 (0.01)	-0.02 (0.01)*	-0.02 (0.01)*	-0.02 (0.01)*
last outcome * entropy	-0.03 (0.01)**	-0.03 (0.01)**	-0.03 (0.01)**	-0.03 (0.01)**
last outcome * AH	0.2 (0.1)**	0.2 (0.1)**	0.2 (0.1)**	0.2 (0.1)**
last outcome * PH	-0.02 (0.01)	-0.02 (0.01)	-0.02 (0.01)	-0.02 (0.01)
Vmax * entropy	0.004 (0.005)	0.005 (0.005)	0.004 (0.005)	0.005 (0.005)
Vmax * AH	-0.05 (0.02)	-0.04 (0.02)	-0.05 (0.02)	-0.05 (0.02)
Vmax * PH	-0.003 (0.005)	-0.003 (0.005)	-0.003 (0.005)	-0.003 (0.005)
Entropy * AH	0.02 (0.02)	0.02 (0.02)	0.03 (0.03)	0.03 (0.03)
Entropy * PH	-0.01 (0.005)*	-0.01 (0.005)*	-0.01 (0.01)*	-0.01 (0.01)*
AH * PH	0.01 (0.1)	0.01 (0.1)	0.01 (0.1)	0.04 (0.1)

-1/trial * CEVR		0.02 (0.04)	0.01 (0.04)	0.01 (0.04)
-1/trial * DEV		0.02 (0.03)	0.02 (0.03)	0.02 (0.03)
-1/trial * IEV		0.03 (0.03)	0.03 (0.03)	0.03 (0.03)
AH * CEVR		0.03 (0.2)	0.05 (0.2)	0.005 (0.2)
AH * DEV		-0.02 (0.2)	-0.02 (0.2)	-0.01 (0.2)
AH * IEV		0.2 (0.2)	0.2 (0.2)	0.2 (0.2)
PH * CEVR		0.05 (0.04)	0.05 (0.04)	0.05 (0.04)
PH * DEV		0.05 (0.03)	0.05 (0.03)	0.04 (0.03)
PH * IEV		0.04 (0.03)	0.04 (0.03)	0.05 (0.03)
AH * uncertainty			-0.1 (0.03)	-0.1 (0.03)
PH * uncertainty			0.001 (0.01)	0.001 (0.01)
AH * mean entropy				-1.1 (0.7)
AH * mean Vmax				-0.01 (0.01)
PH * mean entropy				0.03 (0.1)
PH * mean Vmax				0.001 (0.001)
RT(t-1) * last outcome * AH	0.1 (0.05)	0.1 (0.05)	0.1 (0.05)	0.1 (0.05)
**RT(t-1) * last outcome * PH**	**0.1 (0.01)***	**0.1 (0.01)***	**0.1 (0.01)***	**0.1 (0.01)***
**-1/trial * RT(Vmax) * AH**	**0.1 (0.05)**	**0.1 (0.05)**	**0.1 (0.05)**	**0.1 (0.05)**
-1/trial * RT(Vmax) * PH	0.01 (0.01)	0.01 (0.01)	0.01 (0.01)	0.01 (0.01)
-1/trial * CEVR * AH		-0.2 (0.2)	-0.2 (0.2)	-0.2 (0.2)
-1/trial * DEV * AH		-0.1 (0.1)	-0.1 (0.1)	-0.1 (0.1)
-1/trial * IEV * AH		-0.1 (0.1)	-0.1 (0.1)	-0.1 (0.1)
-1/trial * CEVR * PH		-0.02 (0.03)	-0.02 (0.03)	-0.02 (0.03)
-1/trial * DEV * PH		-0.02 (0.03)	-0.02 (0.03)	-0.02 (0.03)
-1/trial * IEV * PH		-0.01 (0.03)	-0.004 (0.03)	-0.005 (0.03)
Constant	0.1 (0.03)**	0.1 (0.05)*	0.1 (0.05)	-0.5 (0.2)**
Observations	27,253	27,253	27,253	27,253
Akaike Inf. Crit.	61,516.6	61,487.9	61,496.5	61,520.9

* p<0.05; ** p<0.01; *** p<0.001

Supplementary Table 5 legend. Regression coefficient estimate (standard error). Effects of interest are bolded. Exact *p* values for effects of interest in the main analysis can be found in the main Results section (Posterior hippocampal responses to local reinforcement (prediction errors) promote exploration; Anterior hippocampal encoding of the global value maximum promotes exploitation).

Supplementary Table 6. Behavioral effects of hippocampal signals: main and sensitivity analyses, replication session.

	Dependent variable:			
	RT			
	Main analysis	+ contingency	+ choice uncertainty	+ subject-level performance
	(1)	(2)	(3)	(4)
-1/trial	0.1 (0.01)***	0.02 (0.04)	0.1 (0.04)*	0.1 (0.04)*
RT(t-1)	0.5 (0.01)***	0.5 (0.01)***	0.5 (0.01)***	0.5 (0.01)***
RT(Vmax, t-1)	0.1 (0.01)***	0.1 (0.01)***	0.1 (0.01)***	0.1 (0.01)***
last outcome: omission vs. reward	-0.1 (0.01)***	-0.1 (0.01)***	-0.1 (0.01)***	-0.1 (0.01)***
Vmax, within-subject	0.001 (0.01)	0.01 (0.01)	0.01 (0.01)	0.01 (0.01)
entropy, within-subject	0.04 (0.01)***	0.04 (0.01)***	0.03 (0.01)***	0.03 (0.01)***
AH low entropy resp.	0.04 (0.1)	0.3 (0.2)	0.3 (0.2)	1.0 (0.9)
PH RPE resp.	-0.01 (0.03)	0.03 (0.04)	0.04 (0.04)	0.1 (0.2)
contingency: CEVR vs. CEV		-0.05 (0.05)	-0.05 (0.05)	-0.1 (0.05)
contingency: DEV vs. CEV		-0.2 (0.04)***	-0.2 (0.04)***	-0.2 (0.04)***
contingency: IEV vs. CEV		0.1 (0.04)**	0.1 (0.04)**	0.1 (0.04)**
uncertainty of last choice			0.04 (0.01)***	0.04 (0.01)***
mean entropy, between-subjects				0.3 (0.2)
mean Vmax, between-subjects				0.005 (0.001)**
-1/trial * RT(t-1)	-0.003 (0.01)	-0.01 (0.01)	-0.004 (0.01)	-0.004 (0.01)
-1/trial * last outcome	0.05 (0.01)***	0.04 (0.01)***	0.04 (0.01)***	0.04 (0.01)***
-1/trial * RT(Vmax)	0.02 (0.02)	0.01 (0.02)	0.01 (0.02)	0.01 (0.02)
-1/trial * Vmax	-0.003 (0.01)	-0.01 (0.01)**	-0.01 (0.01)**	-0.02 (0.01)**
-1/trial * entropy	-0.003 (0.01)	-0.003 (0.01)	-0.003 (0.01)	-0.003 (0.01)
-1/trial * AH	0.03 (0.04)	0.02 (0.2)	0.04 (0.2)	0.04 (0.2)
-1/trial * PH	-0.01 (0.01)	0.003 (0.04)	-0.004 (0.04)	-0.004 (0.04)
RT(t-1) * RT(Vmax)	-0.02 (0.005)***	-0.02 (0.005)***	-0.01 (0.005)**	-0.02 (0.005)***
RT(t-1) * last outcome	-0.4 (0.01)***	-0.4 (0.01)***	-0.4 (0.01)***	-0.4 (0.01)***
RT(t-1) * Vmax	-0.02 (0.01)**	-0.02 (0.01)**	-0.02 (0.01)**	-0.02 (0.01)**
RT(t-1) * entropy	0.04 (0.01)***	0.04 (0.01)***	0.04 (0.01)***	0.04 (0.01)***
RT(t-1) * AH	0.02 (0.04)	0.01 (0.04)	0.02 (0.04)	0.02 (0.04)
**RT(t-1) * PH**	**-0.04 (0.01)*****	**-0.04 (0.01)*****	**-0.04 (0.01)*****	**-0.04 (0.01)*****
RT(Vmax) * last outcome	0.1 (0.01)***	0.1 (0.01)***	0.1 (0.01)***	0.1 (0.01)***
RT(Vmax) * Vmax	0.1 (0.01)***	0.1 (0.01)***	0.1 (0.01)***	0.1 (0.01)***
RT(Vmax) * entropy	-0.02 (0.01)***	-0.02 (0.01)***	-0.02 (0.01)***	-0.02 (0.01)***
**RT(Vmax) * AH**	**0.1 (0.03)***	**0.1 (0.03)***	**0.1 (0.03)†**	**0.1 (0.03)***
RT(Vmax) * PH	0.01 (0.01)	0.003 (0.01)	0.003 (0.01)	0.003 (0.01)
last outcome * Vmax	-0.01 (0.01)	-0.01 (0.01)	-0.01 (0.01)	-0.01 (0.01)
last outcome * entropy	-0.04 (0.01)***	-0.04 (0.01)***	-0.04 (0.01)***	-0.04 (0.01)***
last outcome * AH	0.1 (0.05)	0.1 (0.05)	0.1 (0.05)	0.1 (0.05)
last outcome * PH	0.02 (0.01)*	0.02 (0.01)*	0.02 (0.01)*	0.02 (0.01)*
Vmax * entropy	0.001 (0.004)	0.002 (0.004)	0.002 (0.004)	0.002 (0.004)
Vmax * AH	-0.02 (0.02)	-0.02 (0.02)	-0.02 (0.02)	-0.02 (0.02)
Vmax * PH	-0.01 (0.004)	-0.01 (0.004)	-0.01 (0.004)	-0.01 (0.004)
Entropy * AH	0.02 (0.02)	0.02 (0.02)	0.02 (0.02)	0.02 (0.02)
Entropy * PH	-0.001 (0.004)	-0.000 (0.004)	0.002 (0.005)	0.002 (0.005)
AH * PH	-0.004 (0.1)	-0.01 (0.1)	-0.01 (0.1)	-0.01 (0.1)

-1/trial * CEVR		0.1 (0.04)	0.04 (0.05)	0.04 (0.05)
-1/trial * DEV		-0.01 (0.04)	-0.04 (0.04)	-0.04 (0.04)
-1/trial * IEV		0.1 (0.04)*	0.1 (0.04)	0.1 (0.04)
AH * CEVR		-0.3 (0.2)	-0.3 (0.2)	-0.3 (0.2)
AH * DEV		-0.4 (0.2)*	-0.4 (0.2)*	-0.4 (0.2)*
AH * IEV		-0.3 (0.2)	-0.3 (0.2)	-0.3 (0.2)
PH * CEVR		-0.1 (0.04)	-0.1 (0.04)	-0.1 (0.04)
PH * DEV		-0.1 (0.03)*	-0.1 (0.03)*	-0.1 (0.04)*
PH * IEV		-0.03 (0.03)	-0.03 (0.03)	-0.02 (0.04)
AH * uncertainty			-0.01 (0.03)	-0.01 (0.03)
PH * uncertainty			-0.01 (0.01)	-0.01 (0.01)
AH * mean entropy				-0.7 (0.9)
AH * mean Vmax				-0.003 (0.01)
PH * mean entropy				-0.1 (0.2)
PH * mean Vmax				0.001 (0.001)
RT(t-1) * last outcome * AH	0.2 (0.04)***	0.2 (0.04)***	0.2 (0.04)***	0.2 (0.04)***
RT(t-1) * last outcome * PH	0.03 (0.01)***	0.03 (0.01)***	0.03 (0.01)***	0.03 (0.01)***
**-1/trial * RT(Vmax) * AH**	**0.1 (0.04)****	**0.1 (0.04)****	**0.1 (0.04)****	**0.1 (0.04)****
-1/trial * RT(Vmax) * PH	-0.02 (0.01)**	-0.02 (0.01)*	-0.02 (0.01)*	-0.02 (0.01)*
-1/trial * CEVR * AH		0.02 (0.2)	-0.001 (0.2)	0.003 (0.2)
-1/trial * DEV * AH		0.03 (0.2)	0.004 (0.2)	0.01 (0.2)
-1/trial * IEV * AH		-0.02 (0.2)	-0.1 (0.2)	-0.05 (0.2)
-1/trial * CEVR * PH		0.001 (0.04)	0.002 (0.04)	0.002 (0.04)
-1/trial * DEV * PH		-0.03 (0.04)	-0.02 (0.04)	-0.03 (0.04)
-1/trial * IEV * PH		-0.004 (0.04)	-0.004 (0.04)	-0.004 (0.04)
Constant	0.1 (0.03)**	0.1 (0.05)**	0.1 (0.05)*	-0.3 (0.2)
Observations	31,999	31,999	31,999	31,999
Akaike Inf. Crit.	68,325.0	68,240.9	68,233.9	68,265.0

* p<0.05; ** p<0.01; *** p<0.001
† p=.054

Supplementary Table 6 legend. Regression coefficient estimate (standard error). Effects of interest are bolded. Exact *p* values for effects of interest in the main analysis can be found in the main Results section (Posterior hippocampal responses to local reinforcement (prediction errors) promote exploration; Anterior hippocampal encoding of the global value maximum promotes exploitation).

Supplementary Table 7. Response hazard as a function of time-varying value and uncertainty and their interactions with hippocampal signals, mixed-effects Cox model

term	Estimate	Standard error	z statistic	*p* value
RT_{t-1}	-0.491	0.008	-62.521	$< 10^{-15}$
PH	0.071	0.072	0.991	0.322
AH	-0.087	0.087	-0.996	0.319
Trial	-0.288	0.031	-9.299	$< 10^{-15}$
Contingency: CEVR vs. CEV	-0.067	0.024	-2.752	0.006
Contingency: DEV vs. CEV	0.092	0.020	4.564	0.00001
Contingency: IEV vs. CEV	-0.163	0.020	-8.101	$< 10^{-15}$
Uncertainty	-0.339	0.011	-30.901	$< 10^{-15}$
Value	0.292	0.005	58.069	$< 10^{-15}$
RT_{t}-1 * PH	0.043	0.007	6.116	$< 10^{-9}$
RT_{t-1} * AH	-0.021	0.007	-2.929	0.003
Trial * CEVR	-0.005	0.034	-0.160	0.873
Trial * DEV	-0.032	0.028	-1.160	0.246
Trial * IEV	-0.090	0.028	-3.244	0.001
Trial * Uncertainty	-0.026	0.018	-1.411	0.158
Trial * Value	0.042	0.008	4.966	0.000001
PH * Value	-0.017	0.004	-3.952	0.0001
AH * Value	0.037	0.005	8.138	$< 10^{-15}$
AH * Uncertainty	-0.017	0.007	-2.604	0.009
PH * Uncertainty	-0.007	0.007	-1.109	0.268

Supplementary Table 7 Legend. Events, $n = 27435$ (70 participants contributing ≤400 trials each). Coefficients, standard errors and unadjusted 2-sided *p* values from a mixed-effects Cox survival model.

Supplementary Figure 1. Posterior hippocampal responses to reward prediction error: detected in the present study, but not in prior meta-analyses.

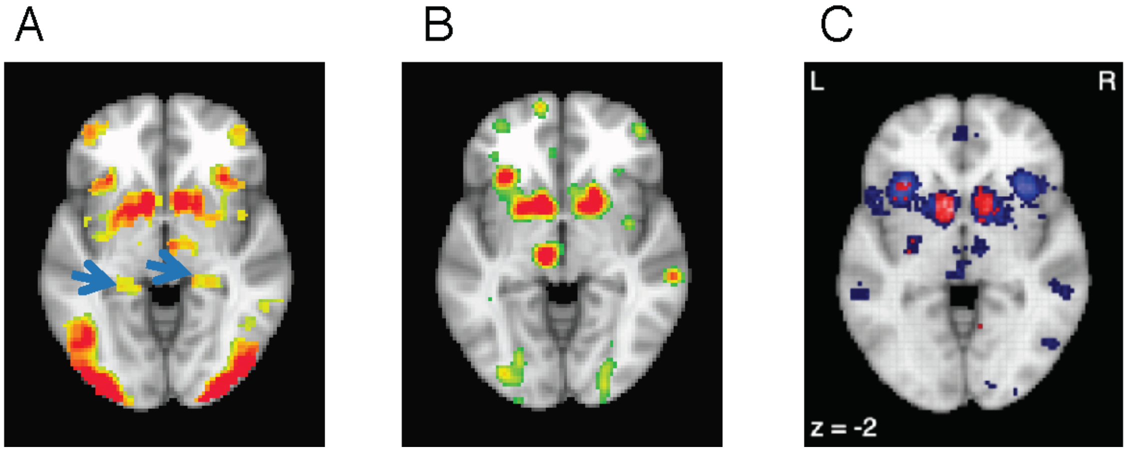

Supplementary Figure 1 caption.

(A) Current study, whole-brain analysis of responses to reward prediction error, $p_{FWE\_corrected} < .05$. Blue arrows: posterior hippocampal clusters. n = 70 participants. Red color corresponds to highest effect of prediction error.

(B). Meta-analysis of studies using reinforcement learning models (Chase et al., 2015). Map liberally thresholded at $p_{uncorrected}<.005$. Red color indicates higher *t*-statistics from the activation likelihood estimation meta-analysis of 38 studies, 751 participants.

(C) Neurosynth, "prediction error", $p_{FDR\_corrected} < .01$. Lighter red color indicates higher *z*-statistics from the association test map. Blue color underlay: lighter blue indicates higher *z*-statistics from the uniformity test. https://neurosynth.org/analyses/terms/prediction%20error/

Supplementary Figure 2. Encoding of reinforcement along the A-P axis when separate regressors are used to model the first half versus second half of each run of the clock task.

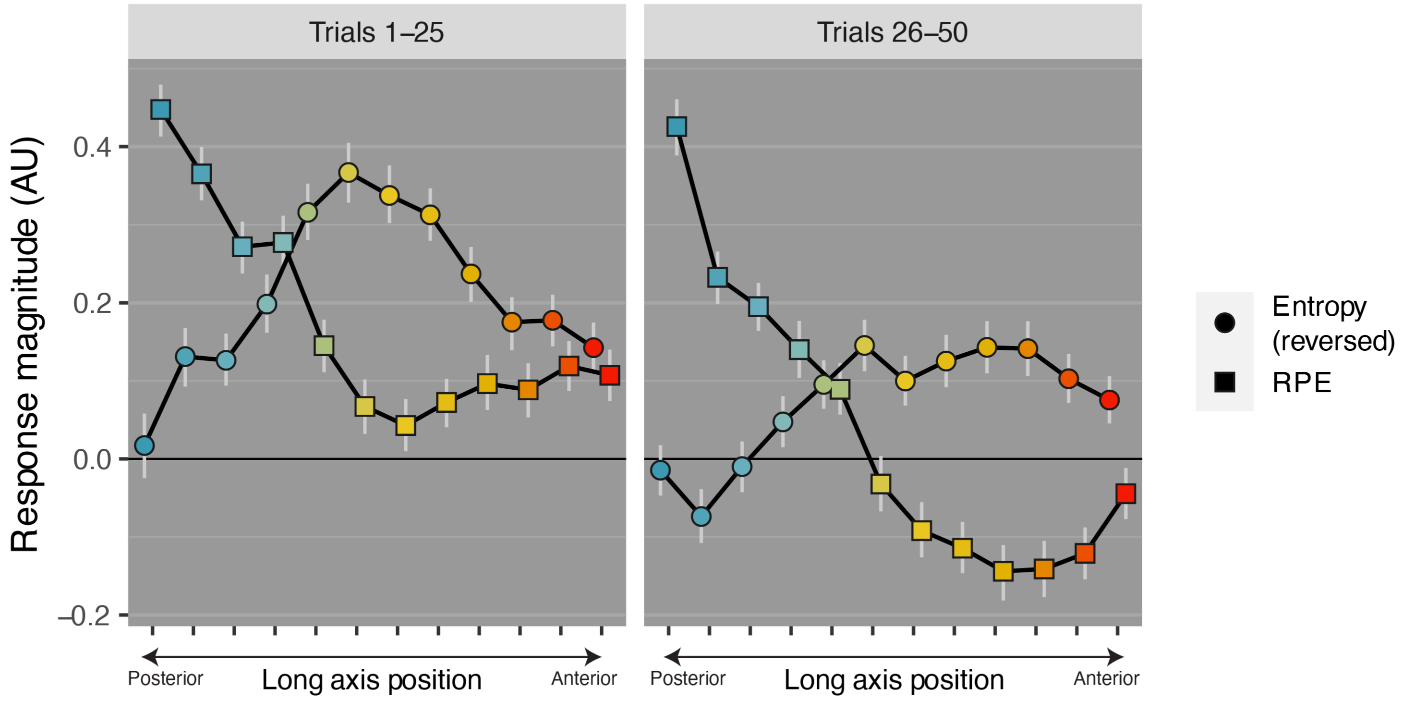

Supplementary Figure 2 Caption.

Double dissociation of signals along the A-P axis when separate model-based regressors were used for the first half (Trials 1-25) versus second half (Trials 26-50) of each run of the clock task. Points represent the mean regression coefficient from voxelwise GLMs along each slice of the hippocampal long axis. The light gray vertical lines denote the bootstrapped 95% confidence interval around the mean. n = 70 participants. Blue: posterior locations, red: anterior locations (see Fig. 2).

Supplementary Figure 3. Hippocampal encoding of immediately preceding response time: sensitivity test for multi-level analysis of deconvolved responses.

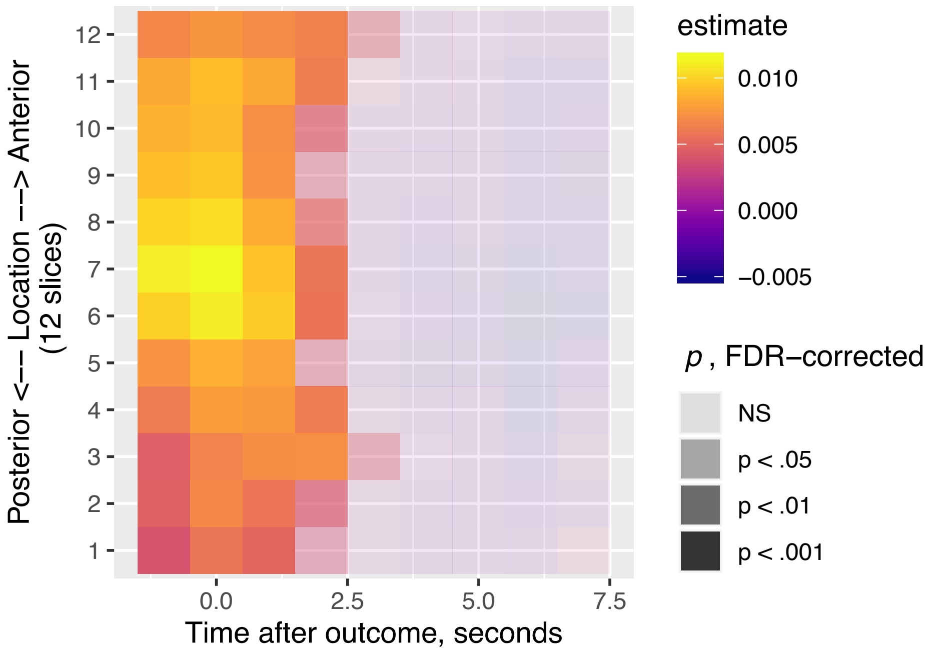

Supplementary Figure 3 caption.

Unfolding hippocampal responses to the preceding response time (before current reinforcement) time-locked to current reinforcement, multi-level general linear model. Positive regression coefficients indicate stronger responses after travelling through a longer part of the interval. n = 70 participants.

Supplementary Figure 4. Encoding of reinforcement along the A-P axis when modeled by simultaneous versus separate model-based decision signals in voxelwise analyses.

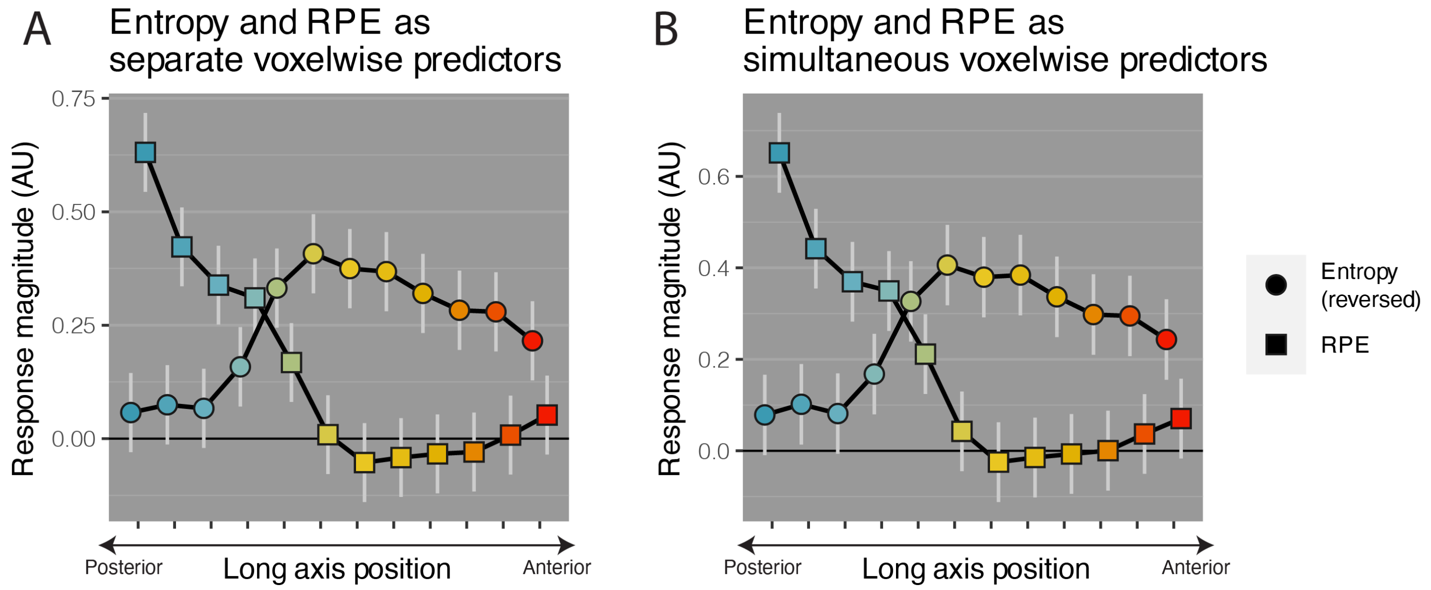

Supplementary Figure 4 caption.

(A) Double dissociation of signals along the A-P axis when Entropy and RPE are entered as model-based fMRI regressors in separate GLMs. Each GLM included the clock and feedback phase regressors. Points denote the average task-related modulation in each hippocampal slice based on voxelwise regression coefficients extracted from GLM analyses. The light gray vertical lines denote the standard error from of the estimated mean from a multilevel regression model. n = 70 participants. Blue: posterior locations, red: anterior locations (see Fig. 2).

(B): Double dissociation of signals along the A-P axis when Entropy and RPE are entered as model-based fMRI regressors in a single GLM. The GLM also included the clock and feedback phase regressors (i.e., 4 regressors total). Points denote the average task-related modulation in each hippocampal slice based on voxelwise regression coefficients extracted from GLM analyses. The light gray vertical lines denote the standard error from of the estimated mean from a multilevel regression model. n = 70 participants. Blue: posterior locations, red: anterior locations (see Fig. 2).

Supplementary Figure 5. Hippocampal online responses to the global value maximum, sensitivity analysis using a restrictive mask developed by Winterburn and colleagues (2013).

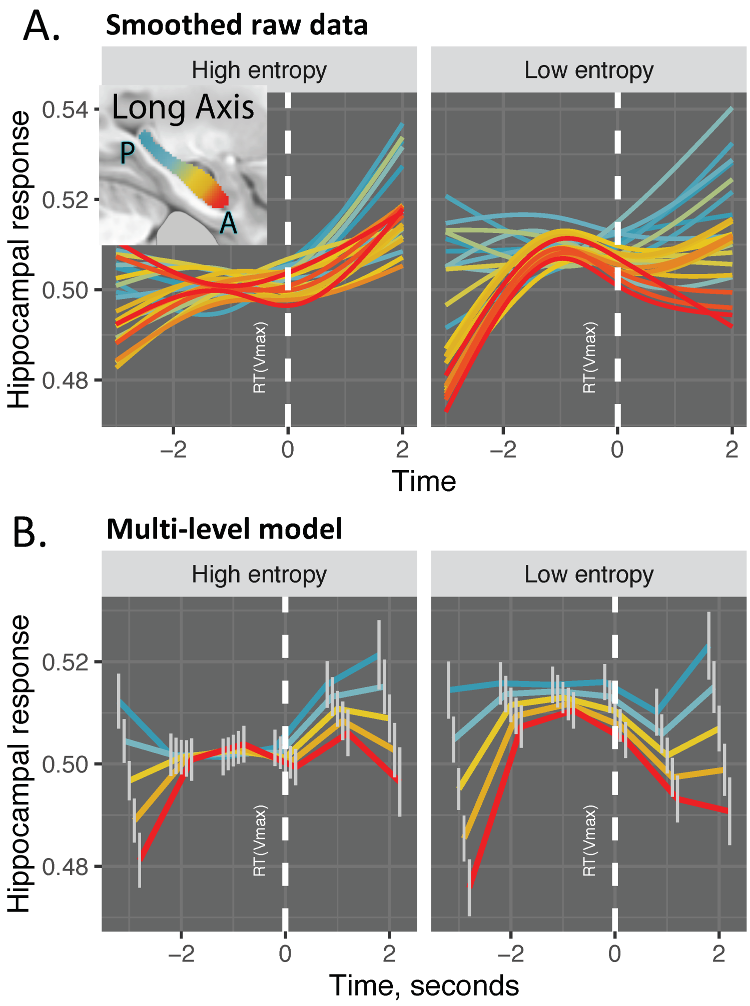

Supplementary Figure 5 caption.

Analyses of deconvolved data. White dashed line: RT_{Vmax}

(A) Raw data, GAM smoothing with 3 knots, voxel-wise responses shown in 24 long-axis bins

(B) Multilevel model, completely general time effect. Event time × location: t = 6.95, $p < 10^{-6}$, event time × entropy: t = 8.33, $p < 10^{-7}$. NB: since voxel-wise timeseries are normalized, only differences in the shape, but not intercept, of response can be interpreted. Centers denote model-estimated mean, vertical gray lines denote standard errors from the multilevel model. n = 70 participants.

Supplementary Figure 6. Hippocampal encoding of reinforcement, sensitivity analysis using a restrictive

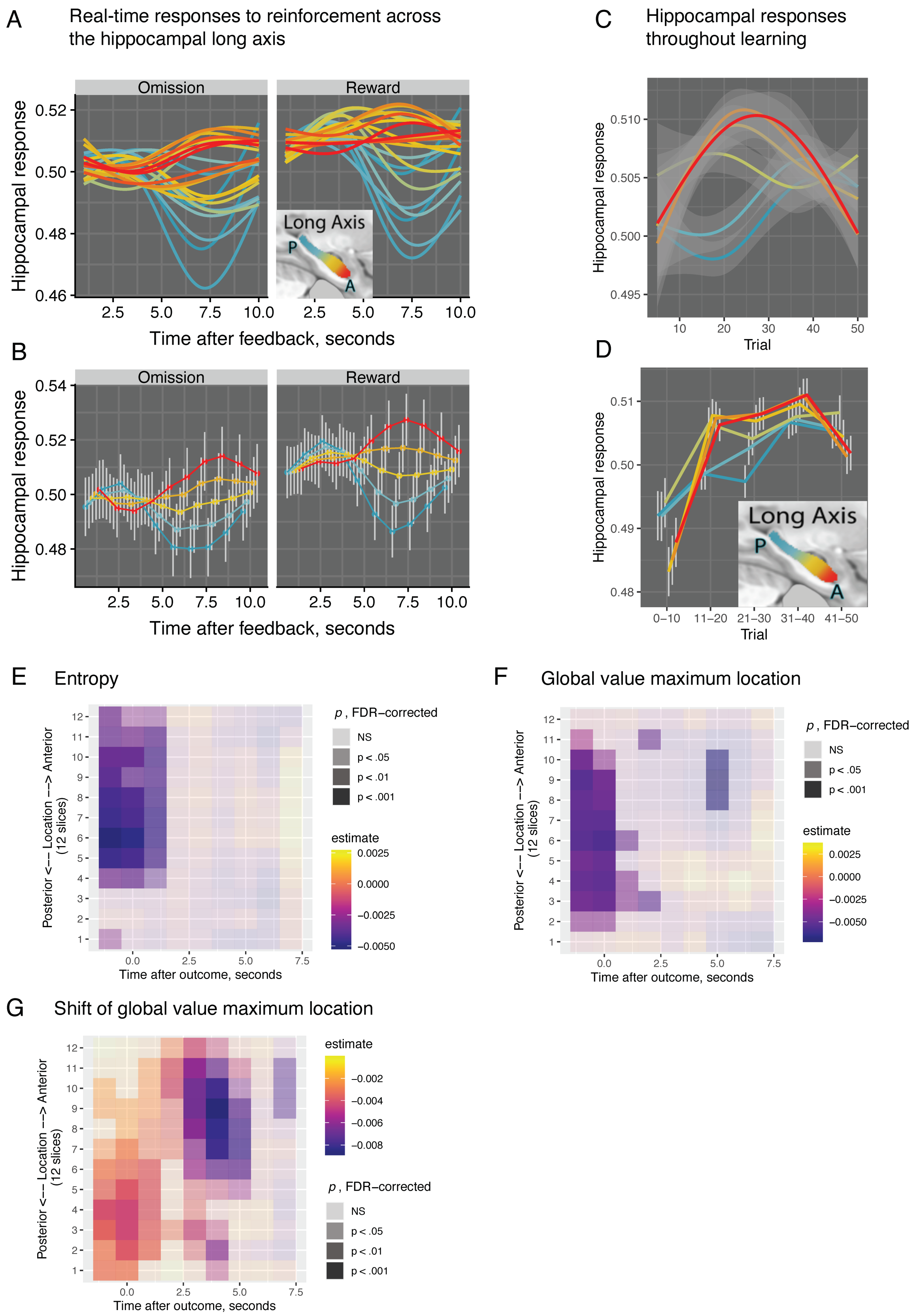

mask.

Supplementary Figure 6 caption.

(A) Responses during the ITI time-locked to feedback, raw data with GAM smoothing, 3 knots.

(B) Responses during the ITI time-locked to feedback, multi-level general linear model with completely general time and bin location (12 bins) as predictors. Centers denote model-estimated mean, vertical gray lines denote standard errors from the multilevel model. n = 70 participants.

(C) Evolution of hippocampal responses to reinforcement across trials, raw data with GAM smoothing, 3 knots. Shaded error bands denote standard errors from the GAM. n = 70 participants.

(D) Evolution of hippocampal responses to reinforcement across trials, multi-level general linear model with completely general *learning epoch* (five 10-trial bins) and bin location (12 bins) as predictors. Centers denote model-estimated mean, vertical gray lines denote standard errors from the multilevel model. n = 70 participants.

(E) Unfolding hippocampal responses to prior entropy (before current reinforcement) time-locked to current reinforcement, multi-level general linear model. Negative regression coefficients indicate stronger responses to low entropy (prominent global value maximum).

(F) Unfolding hippocampal responses to prior global value maximum location (before current reinforcement) time-locked to current reinforcement, multi-level general linear model. Negative regression coefficients indicate stronger responses to a more proximal (earlier) global value maximum.

(G) Unfolding hippocampal responses to prior the shift in the global value maximum location following current reinforcement time-locked to current reinforcement, multi-level general linear model. Negative regression coefficients indicate stronger responses when the global value maximum moves closer (earlier in the interval).
