# Supplementary material for: Differential reinforcement encoding along the hippocampal long axis helps resolve the explore–exploit dilemma
Source: Nat Commun. 2020 Oct 26;11:5407. doi: 10.1038/s41467-020-18864-0 (PMC7589536; doi:10.1038/s41467-020-18864-0)
Supplement: Supplementary file 3 — Reporting Summary [file 41467_2020_18864_MOESM3_ESM.pdf]

nature research

Corresponding author(s): Michael Hallquist

Last updated by author(s): August 17, 2020

Reporting Summary

Nature Research wishes to improve the reproducibility of the work that we publish. This form provides structure for consistency and transparency in reporting. For further information on Nature Research policies, see Authors & Referees and the Editorial Policy Checklist .

Statistics

For all statistical analyses, confirm that the following items are present in the figure legend, table legend, main text, or Methods section.

n/a	Confirmed	
☐	☒	The exact sample size (n) for each experimental group/condition, given as a discrete number and unit of measurement
☐	☒	A statement on whether measurements were taken from distinct samples or whether the same sample was measured repeatedly
☐	☒	The statistical test(s) used AND whether they are one- or two-sided<br>*Only common tests should be described solely by name; describe more complex techniques in the Methods section.*
☐	☒	A description of all covariates tested
☐	☒	A description of any assumptions or corrections, such as tests of normality and adjustment for multiple comparisons
☐	☒	A full description of the statistical parameters including central tendency (e.g. means) or other basic estimates (e.g. regression coefficient) AND variation (e.g. standard deviation) or associated estimates of uncertainty (e.g. confidence intervals)
☐	☒	For null hypothesis testing, the test statistic (e.g. F, t, r) with confidence intervals, effect sizes, degrees of freedom and P value noted<br>*Give P values as exact values whenever suitable.*
☒	☐	For Bayesian analysis, information on the choice of priors and Markov chain Monte Carlo settings
☒	☐	For hierarchical and complex designs, identification of the appropriate level for tests and full reporting of outcomes
☒	☐	Estimates of effect sizes (e.g. Cohen's d, Pearson's r), indicating how they were calculated

Our web collection on statistics for biologists contains articles on many of the points above.

Software and code

Policy information about availability of computer code

Data collection	Collection of behavioral data during the experiment used MATLAB 2012a and PsychToolbox 3.0.10 for stimulus presentation and behavioral measurement.
Data analysis	fMRI analyses used AFNI 19.0.26 and FSL 6.0.1 for processing and general linear modeling. Deconvolution and analysis of hippocampal data depended used open source compiled code and R 3.6.2. All code available here: https://github.com/PennStateDEPENdLab/clock_analysis. Computational models were fit to behavioral data using the open source VBA toolbox and MATLAB 2015a. Code for the computational model is available here: https://github.com/DecisionNeurosciencePsychopathology/temporal_instrumental_agent

For manuscripts utilizing custom algorithms or software that are central to the research but not yet described in published literature, software must be made available to editors/reviewers. We strongly encourage code deposition in a community repository (e.g. GitHub). See the Nature Research guidelines for submitting code & software for further information.

Data

Policy information about availability of data

All manuscripts must include a data availability statement. This statement should provide the following information, where applicable:

- Accession codes, unique identifiers, or web links for publicly available datasets
- A list of figures that have associated raw data
- A description of any restrictions on data availability

The datasets generated during and/or analysed during the current study are available from the corresponding author on reasonable request.

Field-specific reporting

Please select the one below that is the best fit for your research. If you are not sure, read the appropriate sections before making your selection.

☐ Life sciences ☒ Behavioural & social sciences ☐ Ecological, evolutionary & environmental sciences

For a reference copy of the document with all sections, see nature.com/documents/nr-reporting-summary-flat.pdf

Behavioural & social sciences study design

All studies must disclose on these points even when the disclosure is negative.

Study description	Cross-sectional quantitative experimental design.
Research sample	Participants were 70 typically developing adolescents and young adults aged 14–30 (M = 21.4, SD = 5.1). Thirty-seven (52.8%) participants were female and 33 were male. Prior to enrollment, participants were interviewed to verify that they had no history of neurological disorder, brain injury, pervasive developmental disorder, or psychiatric disorder (in self or first-degree relatives). Participants and/or their legal guardians provided informed consent or assent prior to participation in this study. This sample design was chosen to provide a representative sample of typically developing community-dwelling adolescents and young adults.
Sampling strategy	Participants were enrolled based on responses to advertisements in the community or a public research registry. The total sample size was chosen based effect size measures during cognitive tasks using prior developmental cognitive neuroscience fMRI experiments available within the lab.
Data collection	Data were collected using a computer running Windows 7, MATLAB 2012a, and PsychToolbox 3.0.10 for display of the experimental task. Participants completed the task during an fMRI scan and responded using a button glove. Stimuli were displayed in the scanner via a projector screen and a mirror mounted on the head coil. Scans were attended by one research assistant and an MR technician. Neither staff person knew the hypotheses underlying the experimental task.
Timing	Start: 11/19/2013; Stop: 4/25/2015
Data exclusions	Five participants who completed the fMRI scan were excluded from analyses. One was found to have a prior ADHD diagnosis and fell below our intellectual screening threshold (IQ screening < 80). One had reconstruction problems with the MRI data that rendered the scans invalid. Two participants showed virtually no response time variability and did not try later response times; this lack of variability did not allow for learning of the task. One participant had remarkably large head movements (20mm or greater in more than one run). Prior to enrollment, participants were interviewed to verify that they had no history of neurological disorder, brain injury, pervasive developmental disorder, or psychiatric disorder (in self or first-degree relatives).
Non-participation	No participants dropped out of the study after initial enrollment.
Randomization	There was no randomization of participants to groups. This was a single-group normative study.

Reporting for specific materials, systems and methods

We require information from authors about some types of materials, experimental systems and methods used in many studies. Here, indicate whether each material, system or method listed is relevant to your study. If you are not sure if a list item applies to your research, read the appropriate section before selecting a response.

Materials & experimental systems

n/a	Involved in the study	
☒	☐	Antibodies
☒	☐	Eukaryotic cell lines
☒	☐	Palaeontology
☒	☐	Animals and other organisms
☐	☒	Human research participants
☒	☐	Clinical data

Methods

n/a	Involved in the study	
☒	☐	ChIP-seq
☒	☐	Flow cytometry
☐	☒	MRI-based neuroimaging

Human research participants

Policy information about studies involving human research participants

Population characteristics	See above.
Recruitment	Participants were recruited from the larger Pittsburgh area using flyers, electronic advertisements, and a public research registry. This convenience sampling approach may have over-recruited affluent or White participants, though study personnel attempted to correct for this by soliciting participants from public school settings and by over-enrolling ethnic minority participants when possible (consistent with the recruitment plan of the parent NIMH grant).
Ethics oversight	University of Pittsburgh Institutional Review Board

Note that full information on the approval of the study protocol must also be provided in the manuscript.

Magnetic resonance imaging

Experimental design

Design type	Fast event-related fMRI design
Design specifications	Each participant completed 8 blocks of the task consisting of 50 trials each. The decision phase of each trial was up to 4s (could be less if participants responded more quickly) and the feedback phase was 900ms. The intertrial interval varied between 1 and 11 seconds, following an exponential distribution, with the distribution of ITIs chosen to minimize correlations among regressors using Monte Carlo simulations
Behavioral performance measures	The primary performance measure during the task was participant's response times, reflecting their choice about when in time to respond. As described in the paper, participants were rewarded according to a time-varying contingency.

Acquisition

Imaging type(s)	functional imaging for primary analyses; T1-weighted anatomical scan used for nonlinear warping to the MNI template
Field strength	3T
Sequence & imaging parameters	Simultaneous multislice echoplanar imaging (5x multiband acceleration), TR = 1s; TE = 30ms; FoV = 220mm in-plane; 2.3mm isocubic voxel size.
Area of acquisition	Whole-brain acquisition

Diffusion MRI ☐ Used ☒ Not used

Preprocessing

Preprocessing software	Functional images were preprocessed using tools from NiPy, AFNI (version 19.0.26), and the FMRIB software library (FSL version 6.0.1). First, slice timing and motion coregistration were performed simultaneously using a four-dimensional registration algorithm implemented in NiPy. Non-brain voxels were removed from functional images by masking voxels with low intensity and by a brain extraction algorithm implemented in the program ROBEX. We reduced distortion due to susceptibility artifacts using fieldmap correction implemented in FSL FUGUE.<br><br>The participants’ functional images were aligned to their anatomical scan using the white matter segmentation of each image and a boundary-based registration algorithm, augmented by fieldmap unwarping coefficients. Given the low contrast between gray and white matter in echoplanar scans with fast repetition times, we first aligned functional scans to a single-band fMRI reference image with better contrast. The reference image was acquired using the same scanning parameters, but without multiband acceleration. Functional scans were then warped into MNI152 template space (2.3mm resolution) in one step using the concatenation of functional-reference, fieldmap unwarping, reference-structural, and structural-MNI152 transforms. Images were spatially smoothed using a 5mm full-width at half maximum (FWHM) kernel using a nonlinear smoother implemented in FSL SUSAN. Whereas all voxels were spatially smoothed in our whole-brain analyses, our detailed analyses of hippocampal timecourses used a 5mm FWHM smoother within the anatomical mask to reduce partial volume effects (details below). To reduce head motion artifacts, we then conducted an independent component analysis for each run using FSL MELODIC. The spatiotemporal components were then passed to a classification algorithm, ICA-AROMA, validated to identify and remove motion-related artifacts. Components identified as noise were regressed out of the data using FSL regfilt (non-aggressive regression approach). ICA-AROMA has performed very well in head-to-head comparisons of alternative strategies for reducing head motion artifacts. We then applied a .008 Hz temporal high- pass filter to remove slow-frequency signal changes; the same filter was applied to all regressors in GLM analyses. Finally, we renormalized each voxel time series to have a mean of 100 to provide similar scaling of voxelwise regression coefficients across runs and participants.
Normalization	Anatomical scans were registered to the MNI152 2009c template using both affine (ANTS SyN) and nonlinear (FSL FNIRT) transformations. Functional scans were then warped into MNI152 template space (2.3mm resolution) in one step using the concatenation of functional-reference, fieldmap unwarping, reference- structural, and structural-MNI152

transforms.

Normalization template | MNI152 template space, 2009c release from Montreal Neurological Institute.

Noise and artifact removal | Motion-related artifacts were identified and regressed out using the ICA-AROMA package (Pruim 2015). We further included average white matter and CSF voxels and their derivatives as nuisance regressors in voxelwise GLMs.

Volume censoring | Volume censoring was not applied. Rather, ICA-AROMA was used to regress out spatiotemporal components associated with motion. This approach has been demonstrated to be more effective in reducing motion-related artifacts while preserving temporal degrees of freedom (Ciric et al., 2017, NeuroImage).

Statistical modeling & inference

Model type and settings | Whole-brain fMRI analyses were conducted using voxelwise mass univariate general linear modeling in FSL 6.0.1. We used FILM GLS with prewhitening (Tukey tapering of the autocorrelation function) at the run level and FLAME 1+2 for multilevel random effects modeling at the group level. Regression coefficients for subjects were extracted for further analysis. In addition, we used custom analyses of hippocampal activity based on anatomical masks of this region.

Effect(s) tested | GLM design matrices included onset of the decision phase (clock), feedback, and regressors from the SCEPTIC computational model. In particular, we focused on two parametric modulators, reward prediction error (aligned to feedback onset) and entropy of the value distribution (choice phase).

Specify type of analysis: ☐ Whole brain ☐ ROI-based ☒ Both

Anatomical location(s) | Harvard-Oxford probabilistic atlas for subcortical structures (hippocampus). CoBrA atlas for hippocampus using the method of Winterburn et al., 2013. Both masks were based on anatomical segmentation approaches.

Statistic type for inference
(See Eklund et al. 2016) | For whole-brain analyses, we used a voxelwise threshold of $p < .001$ and cluster $p < .05$. To avoid the pitfalls of a parametric (e.g., Gaussian) assumption on the autocorrelation function, we conducted Monte Carlo simulations of cluster significance using permutation of the residuals from the first-level GLM models, consistent with Eklund and colleagues (see also Cox 2018).

Correction | Whole-brain corrections were based on Monte Carlo simulations (using 3dttest++ -Clustsim) based on permutation of the GLM residuals. Slice x time analyses of the hippocampus were FDR-corrected using the method of Benjamini and Yekutieli.

Models & analysis

n/a	Involved in the study
☒	☐ Functional and/or effective connectivity
☒	☐ Graph analysis
☒	☐ Multivariate modeling or predictive analysis
